# Time to establish an international vaccine candidate pool for potential highly infectious respiratory disease: a community’s view

**DOI:** 10.1016/j.eclinm.2023.102222

**Published:** 2023-09-26

**Authors:** Lan Yao, Hiam Chemaitelly, Emanuel Goldman, Esayas Kebede Gudina, Asma Khalil, Rahaman Ahmed, Ayorinde Babatunde James, Anna Roca, Mosoka Papa Fallah, Andrew Macnab, William C. Cho, John Eikelboom, Farah Naz Qamar, Peter Kremsner, Miquel Oliu-Barton, Ivan Sisa, Birkneh Tilahun Tadesse, Florian Marks, Lishi Wang, Jerome H. Kim, Xia Meng, Yongjun Wang, Alyce D. Fly, Cong-Yi Wang, Sara W. Day, Scott C. Howard, J. Carolyn Graff, Marcello Maida, Kunal Ray, Carlos Franco-Paredes, Tapfumanei Mashe, Ngashi Ngongo, Jean Kaseya, Nicaise Ndembi, Yu Hu, Maria Elena Bottazzi, Peter J. Hotez, Ken J. Ishii, Gang Wang, Dianjun Sun, Lotfi Aleya, Weikuan Gu

**Affiliations:** a Department of Nutrition and Health Science, College of Health, Ball State University, Muncie, IN 47306, USA; b Department of Orthopedic Surgery and BME-Campbell Clinic, University of Tennessee Health Science Centre, Memphis, TN 38163, USA; c Infectious Disease Epidemiology Group, Weill Cornell Medicine-Qatar, Cornell University, Doha, Qatar; d World Health Organization Collaborating Centre for Disease Epidemiology Analytics on HIV/AIDS, Sexually Transmitted Infections, and Viral Hepatitis, Weill Cornell Medicine–Qatar, Cornell University, Qatar Foundation – Education City, Doha, Qatar; e Department of Population Health Sciences, Weill Cornell Medicine, Cornell University, New York, NY, USA; f Department of Microbiology, Biochemistry and Molecular Genetics, New Jersey Medical School, Rutgers University, Newark, NJ 07103, USA; g Department of Internal Medicine, Jimma University Institute of Health, Jimma, Ethiopia; h Fetal Medicine Unit, St George’s Hospital, St George’s University of London, London, UK; i Cell Biology and Genetics Department, University of Lagos, Lagos 101017, Nigeria; j Centre for Human Virology and Genomics, Microbiology Department, Nigerian Institute of Medical Research, Lagos 100001, Nigeria; k Department of Biochemistry and Nutrition, Nigerian Institute of Medical Research, Yaba, Lagos State, Nigeria; l Medical Research Council Unit, The Gambia at the London School of Hygiene and Tropical Medicine, Fajara 273, The Gambia; m Refuge Place International, Monrovia, Liberia; n The Stellenbosch Institute for Advanced Study (STIAS), Wallenberg Research Centre at Stellenbosch University, South Africa; o Department of Clinical Oncology, Queen Elizabeth Hospital, Kowloon, Hong Kong, China; p Population Health Research Institute, McMaster University and Hamilton Health Sciences Hamilton, Canada; q Department of Pediatrics and Child Health, Aga Khan University Hospital, National Stadium Rd, Karachi, Sindh 74800, Pakistan; r Institut für Tropenmedizin, Universität Tübingen, Germany; s Université Paris Dauphine – PSL, Pl. du Maréchal de Lattre de Tassigny, Paris 75016, France; t Bruegel, Rue de la Charité 33, Brussels 1210, Belgium; u College of Health Sciences, Universidad San Francisco de Quito, Quito 170901, Ecuador; v International Vaccine Institute, Seoul, Republic of Korea; w Department of Basic Medicine, Inner Mongolia Medical University, Jinshan Development Zone, Huhhot, China; x International Vaccine Institute, Seoul, Republic of Korea; y Seoul National University, College of Natural Sciences, Seoul, Republic of Korea; z Department of Neurology, Beijing Tiantan Hospital, Capital Medical University, Beijing 100050, China; aa NHC Key Laboratory of Respiratory Diseases, Department of Respiratory and Critical Care Medicine, The Centre for Biomedical Research, Tongji Hospital, Tongji Medical College, Huazhong University of Science and Technology, Wuhan 430030, China; ab College of Nursing, University of Tennessee Health Science Center, Memphis, TN 38105, USA; ac Gastroenterology and Endoscopy Unit, S. Elia-Raimondi Hospital, Caltanissetta 93100, Italy; ad School of Biological Science, Ramkrishna Mission Vivekananda Education & Research Institute, Narendrapur 700103, West Bengal, India; ae Hospital Infantil de Mexico, Federico Gomez, Mexico; af One Health Office, Ministry of Health and Child Care, Harare, Zimbabwe; ag World Health Organization, Harare, Zimbabwe; ah Institute of Human Virology, Abuja, Nigeria; ai Institute of Haematology, Union Hospital, Tongji Medical College, Huazhong University of Science and Technology, 1277 Jiefang Avenue, Wuhan 430022, China; aj Hubei Clinical and Research Centre of Thrombosis and Hemostasis, Wuhan, China; ak Department of Pediatrics, Texas Children's Hospital Centre for Vaccine Development, Baylor College of Medicine, Houston, TX, USA; al National School of Tropical Medicine, Baylor College of Medicine, Houston, TX, USA; am Molecular Virology & Microbiology, Baylor College of Medicine, Houston, TX, USA; an Division of Vaccine Science, Department of Microbiology and Immunology, The Institute of Medical Science, The University of Tokyo, Tokyo, Japan; ao International Vaccine Design Centre, The Institute of Medical Science, The University of Tokyo, Tokyo, Japan; ap Centre for Vaccine Adjuvant Research, National Institutes of Biomedical Innovation, Health and Nutrition, Osaka, Japan; aq Department of Pancreatic and Biliary Surgery, The First Affiliated Hospital of Harbin Medical University, Harbin, China; ar Centre for Endemic Disease Control, Chinese Centre for Disease Control and Prevention, Harbin Medical University; Key Laboratory of Etiologic Epidemiology, Education Bureau of Heilongjiang Province & Ministry of Health 23618104, 157 Baojian Road, Harbin, Heilongjiang 150081, China; as Chrono-Environnement Laboratory, UMR CNRS 6249, Bourgogne Franche-Comté University, Besançon Cedex F-25030, France; at Research Service, Memphis VA Medical Centre, 1030 Jefferson Avenue, Memphis, TN 38104, USA; au Vascular Biology Research Centre, Molecular and Clinical Sciences Research Institute, St George’s University of London, London, UK; av Centre for Emerging Infectious Diseases Policy and Research, Boston University, Boston, MA, USA; aw Africa Centre for Disease Control, Addis Ababa, Ethiopia; ax Department of Medicine, McMaster University, Hamilton, ON, Canada; ay Centre de Recherches Medicales de Lambarene, Gabon; az Department of Microbiology, Immunology, and Pathology, Colorado State University, USA

**Keywords:** COVID-19, Disease, Infection, International collaboration, Vaccine

## Abstract

In counteracting highly infectious and disruptive respiratory diseases such as COVID-19, vaccination remains the primary and safest way to prevent disease, reduce the severity of illness, and save lives. Unfortunately, vaccination is often not the first intervention deployed for a new pandemic, as it takes time to develop and test vaccines, and confirmation of safety requires a period of observation after vaccination to detect potential late-onset vaccine-associated adverse events. In the meantime, nonpharmacologic public health interventions such as mask-wearing and social distancing can provide some degree of protection. As climate change, with its environmental impacts on pathogen evolution and international mobility continue to rise, highly infectious respiratory diseases will likely emerge more frequently and their impact is expected to be substantial. How quickly a safe and efficacious vaccine can be deployed against rising infectious respiratory diseases may be the most important challenge that humanity will face in the near future. While some organizations are engaged in addressing the World Health Organization's "blueprint for priority diseases", the lack of worldwide preparedness, and the uncertainty around universal vaccine availability, remain major concerns. We therefore propose the establishment of an international candidate vaccine pool repository for potential respiratory diseases, supported by multiple stakeholders and countries that contribute facilities, technologies, and other medical and financial resources. The types and categories of candidate vaccines can be determined based on information from previous pandemics and epidemics. Each participant country or region can focus on developing one or a few vaccine types or categories, together covering most if not all possible potential infectious diseases. The safety of these vaccines can be tested using animal models. Information for effective candidates that can be potentially applied to humans will then be shared across all participants. When a new pandemic arises, these pre-selected and tested vaccines can be quickly tested in RCTs for human populations.

## Introduction

Mitigating the impact of an infectious disease pandemic entails the availability of effective vaccines that can reduce disease severity and prevent deaths. By the end of 2022, coronavirus disease 2019 (COVID-19) claimed almost 7 million lives around the world, with the death rate being substantially higher during the first disease wave substantially higher during the first disease wave, prior to vaccine availability^1^, ^2^, ^3^ (https://www.worldometers.info/coronavirus/). In particular, the death rate declined from 5.7% during the first wave to approximately 1.7% during the second and third waves second and third waves in developed countries, when vaccines became available when vaccines became available and the later variants, Omicron and Delta, caused significantly less morbidity and mortality.^4^ Evidence suggests a 60%–90% decline in severity following vaccination,^5^, ^6^, ^7^ and that vaccination remains the safest and best tool for protecting against COVID-19-related hospitalization and death, irrespective of previous infection status.^8^ Once vaccines were available, they averted an estimated 20 million deaths from COVID-19.^9^ Widespread vaccination is also expected to reduce the economic burden of the disease and its impact on social liberties,^10^^,^^11^ and the likelihood of the emergence of immune escape virus variants.^12^, ^13^, ^14^ Among all pandemic diseases, air transmissible respiratory diseases are the most contagious and difficult to contain. The critical question is thus how to make vaccines available as early as possible during future similar or even more severe pandemics.

## Risk of emerging respiratory infectious disease pandemics

The severe acute respiratory syndrome coronavirus 2 (SARS-CoV-2) pandemic is thus far the most contagious and disruptive infectious disease of the 21st century, causing a humanitarian crisis and high mortality. This pandemic is unlikely to be the last. Evidence suggests that the occurrence of such pandemics may become more frequent in the near future.^15^^,^^16^ Thus, according to the WHO and the US CDC there are several pathogens that could cause future pandemics including Crimean-Cong haemorrhagic fever, Ebola virus disease and Marburgh virus disease, Lassa fever, Middle East respiratory syndrome (MERS), Nipah and henipaviral disease, Rift Valley fever, Zika virus, Disease X, Cholera, Polio, Enterovirus-71, high drug-resistant Tuberculosis and avian influenza H5N1.^17^^,^^18^ For example, as this manuscript is written the avian influenza H5N1 is subject of close surveillance by the WHO and the Institute of Public Health of Chile where last March 2023 was reported a human infection of avian influenza H5N1.^19^

Infectious diseases transmission are driven by several factors. Human interaction is one of the main factors at environmental level. For instance, global warming increases the chance of survival of microorganisms and their global spread.^15^^,^^20^ The urbanization phenomenon through new road construction has been associated with higher *R*_*0*_ (reproductive number) for viral, bacterial and protozoan pathogens transmission in nonremote villages compared to remote communities.^21^ In addition, urbanization has caused deforestation and contamination of major watersheds and local climate, both of which can affect the transmission of enteric pathogens.^18^^,^^22^

Modernization and development of human society increases domestic and international travels, contributing to the transmission of infectious diseases in human populations.^23^ Population growth and density also increase exposure to bacteria, viruses and animals, and therefore further infection transmission.^16^ The impact of human and societal development on the spread of infectious disease is hard to mitigate, making infectious diseases an inevitable health challenge that humanity must face.^24^

## The urgent need for an international vaccine candidate repository pool for potential respiratory infectious diseases

Before endorsing a framework that discusses the organization of an international collaboration on vaccine candidates pool, it is imperative to acknowledge that future infectious respiratory diseases threaten all human societies and cannot be contained unless globally controlled. For example, SARS-CoV-2 showed growing resilience and sustainable circulation with emerging variants.^8^ In the future, a new dreadful pandemic that spreads rapidly may emerge. The emergence of new variants of concern within the existing COVID-19 pandemic remains possible as well. Confronting such threats requires the support of not only scientific societies but also governments and public and private organizations around the world. In addition to existing organizations and pharmaceutical companies for vaccine development (Supplementary Table S1), if there were a new pandemic, a centralized data and resource exchange would also be helpful. Providing data on vaccine safety and effectiveness of the potential vaccines would enormously help modeling and forecast, especially in the mid- and long-term.

## Proposed mission of the international vaccine pool (InterVax™)

The mission of international vaccine pool InterVax™ is to build a foundation to accelerate the development of vaccines against potential future respiratory infectious disease pandemic threats so vaccines can be accessible to any people in need.

The scope of the international pool includes vaccines against known viruses, that may potentially lead to high contiguous and catastrophic pandemics, if mutated or transmitted to humans.

The international pool will focus on the collection and analysis of information on known viruses; design and production of potential vaccines; and testing the safety of potential vaccines in vitro and in vivo on animal models.

The InterVax™ is an open platform that provides information all over the world for utilization; users are expected to follow the same principles of the mission of the international pool. InterVax" will be registered as a non-profit independent entity trademark. Members of the entity have access to the pool and own this trademark equally in terms of health equity.

## Suggested function of the international vaccine pool (InterVax™)

### Funding

International vaccine pool accepts all funds from government, public and private, organizations and individuals. No funds should have any additional condition except equal global distribution of the products of InterVax™.

### Independence

International vaccine pool will work closely with WHO and other major vaccine organizations as an independent entity, leveraging its existing infrastructure and contacts in different countries, to achieve its mission (Supplementary Table S1). Accordingly, the initiative will be available to all countries with no discrimination.

### Interactions

International vaccine pool should collaborate with other international and national organizations, so that information for effective candidates that can be potentially applied to humans will then be shared across all participants to ensure the vaccine reaches where it is needed in case new infectious disease emerge. While the international vaccine pool stands as an independent organization, its interaction with existing organizations for vaccine research and production (listed in Supplementary Table S1 as examples) will benefit from potential resources (WHO, CDCs from variety countries), financial support (CDCs, WHO, CEPI, SRC), disease monitoring (ISID, IVI), network information (IFIC, SCARDA, IVI) and technology renovations (BARDA, VAANZ, GIZ, SRC, etc).

## Possibility for the establishment of the international vaccine pool (InterVax™)

Currently, the three major types of vaccines are mRNA vaccines, protein subunit vaccines, and viral vector vaccines.^25^ These are either directly synthesized in the laboratory, as pieces (e.g., spike proteins) of the virus, or a modified virus. The rapid development of biotechnologies and our understanding of genomic components of various organisms enables us to design and produce a variety of molecules including mRNA and proteins through diverse approaches. First, through genomic sequencing, we can extract genome sequences for an array of viruses, other infectious pathogens, and their hosts. Such information enables estimation, to a high degree of reliability, of the potential pathogenic effect of infectious agents on human hosts, as well as projecting and ranking their potential transmission based on information on clustering from previous pandemics. Second, current technologies allow us to design and produce genomic sequences that can be utilized in the production of mRNA or protein sequences that can be used as vaccines.

The span of diversity in knowledge, technological advancement, resources, and methodologies across different countries and regions suggests that a well-organized international collaboration will lead to the production of a large volume of material that can be solicited for potential vaccine development.

Such an initiative would be of global interest, including to economic giants as exemplified by the COVID-19 pandemic that caused significant losses to all economies. During the pandemic, countries globally were racing to produce or procure effective vaccines against SARS-CoV-2 infection to restore normalcy. The short-term urgency also necessitated an unprecedented pouring of resources to make any vaccine available, even with partial efficacy. Additionally, despite significant investment in research and development, no single country has had the capacity to meet the unprecedented global demand for vaccines. The proposed vaccine pool will allow rapid vaccine production at a much lower cost and will equip countries, including economic giants with better preparedness against the economic and health consequences of future pandemics. The vaccine pool is also likely to encourage innovation in vaccine development providing economic giants with an incentive to produce more effective vaccines that can compete in international markets.

One might ask whether it is even possible to launch such an effort, given the enormous stakes for existing economic giants. While it is certainly a concern that some elements of society will seek to use an emerging pandemic as an economic opportunity, we believe that because the stakes are so high, everyone will join forces to combat a common enemy. To this end, proper public dissemination and communication through the press and social media will be instrumental to align everyone in this battle, including the economic giants.

## Expected benefit of the vaccine candidate pool

**Fig. 1:**
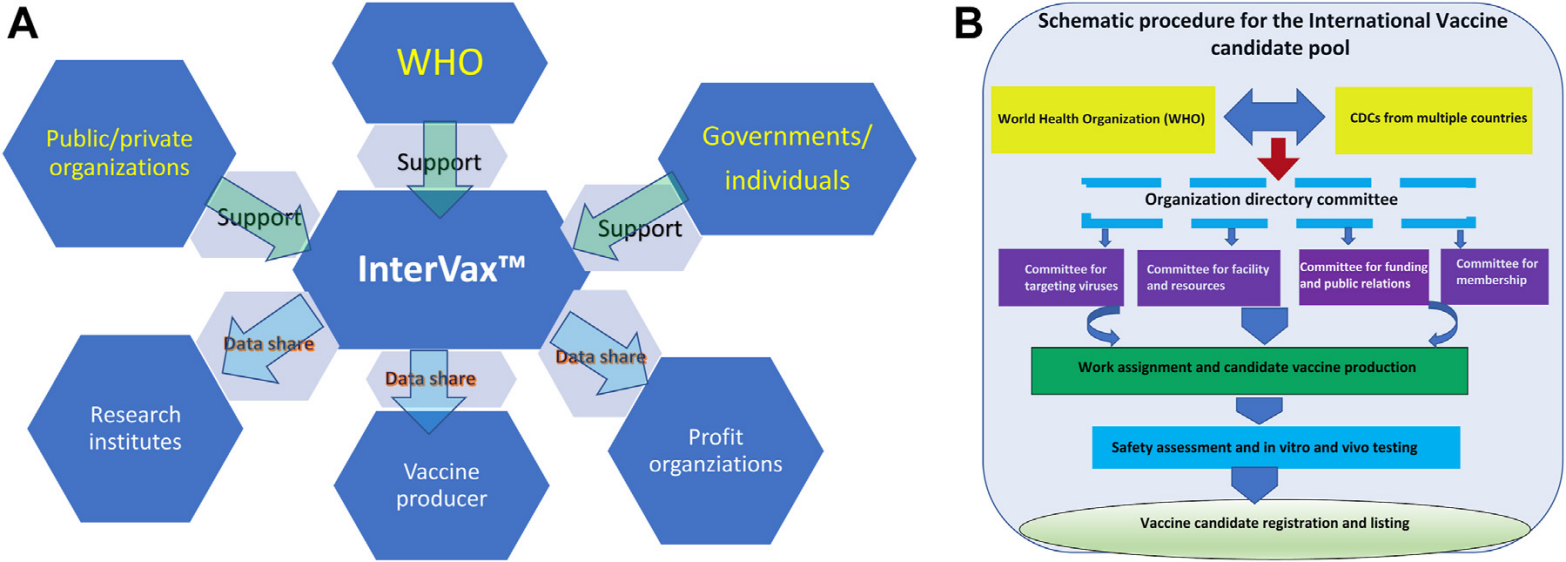
**Function and organization of international vaccine candidate pool.** (A) Function Position and Interaction. It shows how the InterVax works with WHO, Public/Private organizations, Research Institutes, Vaccine producer, Profit organizations and Governments/individuals. (B) Vaccine Pool Organization. The flowchart demonstrates the supports InterVax obtained from various organizations and data shared with industrial sections.

An international vaccine pool could reduce harmful competition among countries or vaccine production companies that emphasize outcome rather than value and expense (Fig. 1A). Outcome-centered competition may lead to all sorts of poor decisions. In addition, the vaccine pool will enhance the detection of repetitive products and therefore alert stakeholders to potential duplication of effort and waste of resources in the event when the same vaccines are studying with the same way in multiple places.

An international vaccine pool may facilitate the production of candidates for all types of vaccines that are ready to be tested and utilized against a new pathogen, notably in case of a new pandemic, whereas an individual country may have limited technologies and resources to develop vaccine candidates for every type of potential infectious virus. Cooperation across countries and regions will thus bring economies of scale.

This system has the potential to protect geographic areas not yet affected by an emerging pandemic. Private sector will likely have a role in a global response, probably in partnership with governments. This was certainly the case with COVID-19 and vaccine development by both Pfizer-BioNTech and Moderna, with the support of the United States and other governments. Again, we are all in this together, and as business and government entities recognize the common threat, the imperative of a common response will take precedence.

An international vaccine pool can be used to ensure timely control of infection spread in areas with emerging variants. It could be used to quickly identify geographical areas where a new disease is emerging and provide available measures to restrict its spread. Without an international collaboration pool, the vaccine may not be available in places where the disease occurs first, or in a large pandemic area. Such places may be in a low-income country where the capability of vaccine production is either weak or even non-existent. An example is monkeypox which is endemic in some African countries but became significant (multicentre drug and vaccine trials) when it affected high-income countries.^26^ Even in areas where new mutants or disease outbreaks have not yet occurred, the vaccine pool could still be beneficial by enabling more rapid and efficient vaccine development and distribution in case of an arising public health emergency. With such an international organization, a vaccine may be available for these places that are in need, notably the regions that are closely connected to where the outbreak was detected, and low-income countries where the spread can lead to more hazard.^27^^,^^28^ Thus, the vaccine pool could address health equity issues by prioritizing the most vulnerable regions. Governments have a vital role to play in supporting the private sector in vaccine development, providing the necessary funding, regulatory support, and incentives to encourage innovation. However, it is crucial to ensure that these efforts align with the broader mission of the vaccine pool of improving global health outcomes and increasing vaccines' availability and accessibility. Governments must prioritize public health and ensure that any private sector involvement is subject to appropriate oversight, transparency, and ethical considerations.

An international vaccine pool can allow pre-testing of the safety and immunogenicity of molecules in animal models. In this case, it saves the time to get into clinical trials of human populations in case infectious disease occurs. In the case of a disease with high infectivity and mortality, saving time means saving hundreds of thousands of lives.

An established platform of an international pool can be utilized to test and document human safety in various populations in different categories to ensure their safe usage. For example, testing the safety and dosages in pregnant women or ensuring efforts to demonstrate safety in pregnancy, one of the lessons learned from the COVID-19 pandemic.^29^^,^^30^

The vaccine pool may also facilitate testing the potential utility of vaccines developed against one infection in protecting the population against other infections. An example to this end is a meningococcal vaccine recently found to be effective against acquiring gonorrhea infection.

An international vaccine pool will effectively works with existing organizations for vaccine production efficiency and reduce the duplication of novel vaccine given an unknown viral outbreak. For example, the vaccine pool complements the 100 days vaccine mission of CEPI in a more effective way, avoiding unnecessary duplication and waste of research & development resources and time, without any negative effect on the new vaccine safety. While CEPI develops its 100 capacity, the InterVax™ will be capable for the information collection and prediction of future threatening viruses. While the 100 days vaccine mission of CEPI is mainly supported by G7 countries, the international vaccine pool will ensure the developing countries to have an equal opportunity for the pandemic preparations.

## Proposed organization and operation of the global vaccine coordination committee

To achieve such an international vaccine candidate pool (Fig. 1B), we call for international action to immediately 1) start the discussion on how to form an organized committee to plan the pool, 2) invite more WHO members to join our mission and to potentially assume WHO a leading role in the organization and coordination of committee activities, 3) invite more scientists from developing countries, from low or middle-income countries, underserved population and under-resourced groups, to join the InterVax™, and 4) establish dialogues among the scientific community, the WHO, world leaders in other health organizations, and relevant governmental and private organizations.

While the vicissitudes of political and cultural differences do pose a challenge, it is worth noting that even in cases of extreme opposition, countries have continued to cooperate on certain activities. We believe the imperative of mutual survival will take precedence over political divisions. In the ideal, Science is an international enterprise that has no national boundaries. The World Health Organization, with headquarters in Switzerland, would be an appropriate organization to oversee this initiative.

## Future directions

When a consensus is formed, work may begin to 1) form an executive committee for activity coordination, 2) form subcommittees and designate coordinators in countries and regions, 3) develop a vaccine synthesis plan to rank potential risks of various known pathogens, 4) collaborate with industry for potential assistance in the accomplishment of certain goals, and 5) start carefully to coordinate the global task for the division of candidate synthesis.

This also entails:

• Creating a live repository of available technologies and molecules used in existing vaccines, available manufacturing and storage facilities, and infrastructure, as well as available financial schemes that can facilitate vaccine development.
• Mapping of research institutions that can facilitate the conduct of safety test and clinical trials such as the Adjuvant Development Program at the National Institute of Allergy and Infectious Diseases in the USA.
• Creation of an aligned incentive structure that rewards government, private, and academic institutions that participate.
• Determine the locations and establish headquarters and offices in different regions and countries.
• Nominate coordinator/s and representatives from major organizations and/or countries.
• Intellectual property management system. Within the InterVax™ to establish a “patent-free platform pool” so that certain vectors such as Adeno, VSV, MVA, etc., could be made available without IP issues.

In conclusion, we urge the international community, especially the WHO, to start a worldwide coordinated synthesis of vaccines and provide locations, resource supplements, and personnel training. Doing so will help achieve effective infection control and regain functional global and local economies in the event of a future pandemic.

## Contributors

Conceived and designed the experiments: LY, JHK, WG, DS, LA. Performed data searching and collection: LY, WG. Analysed the data: All Authors. Contributed analysis tools: WG, DS, LA. Wrote first draft of manuscript: LY, WG, edited by EG, CG. Revise and approve manuscript: All authors.

## Data sharing statement

All the data are available at the public databases as provided in the manuscript.

## Declaration of interests

Peter Hotez is a co-inventor of a COVID-19 recombinant protein vaccine technology owned by Baylor College of Medicine (BCM) that was recently licensed by BCM non-exclusively and with no patent restrictions to several companies committed to advance vaccines for low- and middle-income countries. The co-inventors have no involvement in license negotiations conducted by BCM. Similar to other research universities, a long-standing BCM policy provides its faculty and staff, who make discoveries that result in a commercial license, a share of any royalty income, according to BCM policy. Jerome H. Kim services as the consultant for SK bioscience and Moderna. Other authors declare no competing financial interests.
