# Supplementary material for: Time to establish an international vaccine candidate pool for potential highly infectious respiratory disease: a community’s view
Source: eClinicalMedicine. 2023 Sep 26;64:102222. doi: 10.1016/j.eclinm.2023.102222 (PMC10550631; doi:10.1016/j.eclinm.2023.102222)
Supplement: Supplementary Table S1 [file mmc1.pdf]

Supplementary Table 1. Major International Organizations/Agencies that Support Vaccine Development.

**Organization/Agency**	**Nature and Focus**	**Headquarters/Country/Region**	**Note**
Korea Centers for Disease Control and Prevention/	Preparing for emerging infectious diseases, securing medical resources during a crisis, activating the emergency response during the crisis, and fortifying capabilities of public health personnel	Korea	By government
International Vaccine Institute	A nonprofit inter-governmental organization established in 1997 at the initiative of the United Nations Development Programme (UNDP), focuses on infectious diseases of global health importance	South Korea-based	
The Coalition for Epidemic Preparedness Innovations (CEPI)	A foundation that takes donations from public, private, philanthropic, and civil society organisations to finance independent research projects to develop vaccines against emerging infectious diseases.	Oslo, Norway	Bill & Melinda Gates Foundation, Government of India
International Society for Infectious Diseases (ISID)	Non-profit organization that monitors infectious diseases on a global scale.	Massachusetts, US.	Solicits donations from the general public, as well as governments, foundations, and the pharmaceutical industry
International Federation of Infection Control (IFIC)	An umbrella organization of societies and associations of healthcare professionals in infection control and related fields worldwide. The goal of IFIC is to minimize the risk of infection within the healthcare setting worldwide through the development of a network of	Arlington, VA, USA	

	infection control organizations for communication, consensus building, education, and sharing expertise		
Strategic Center of Biomedical Advanced Vaccine Research and Development for Preparedness and Response (SCARDA)	Collects and analyzes wide-ranging information on vaccine development that will lead to strategically significant research funding and implements both Programs on R&D of New Generation Vaccine including New Modality Application and Japan Initiative for World-leading Vaccine Research and Development Centers.	Japan	A division of Japan Agency for Medical Research and Development
Chinese Center for Disease Control and Prevention	Focuses national attention on developing and applying disease prevention and control (especially infectious diseases), environmental health, occupational safety and health, health promotion, prevention, and education activities designed to improve the health of the people of the People's Republic of China	China	An institution directly under the National Health Commission, based in Changping District, Beijing, China
Global Alliance for Vaccines and Immunization	Public-private global health partnership with the goal of increasing access to immunization in poor countries	Geneva, Switzerland	
German Agency for International Cooperation (GIZ)	An international development agency owned by the German Federal Government. GIZ implements technical cooperation projects of the Federal Ministry for Economic Cooperation and Development	Germany	

Swedish Research Council (SRC)	Sweden's largest governmental research funding body. Supports research of the highest quality within all scientific fields	Sweden	
Biomedical Advanced Research and Development Authority (BARDA)	Its vaccines & immunizations includes vaccines against viruses/infectious diseases	USA	An important part of U.S. Department of Health and Human Services (HHS)
Vaccine Alliance Aotearoa New Zealand – Ohu Kaupare Huaketo (VAANZ)	A nationwide alliance to secure a vaccine for New Zealand	New Zealand	
